# The value of Continuous Remote Monitoring in Clinical Decision Making After Same-day Discharge Metabolic Bariatric Surgery

**DOI:** 10.1007/s11695-026-08729-8

**Published:** 2026-06-11

**Authors:** Anne-Jet S. Jansen, Claudia Berends, Sjoerd H. Garssen, Laura N. Deden, Laura Kooij, Wim H. van Harten, Eric J. Hazebroek, Carine J. M. Doggen

**Affiliations:** 1 Department of Innovation & Care transformation, Rijnstate Hospital, Arnhem, Netherlands; 2 Health Technology and Services Research, Faculty of Behavioral, Management and Social Sciences, Technical Medical Centre, University of Twente, Enschede, Netherlands; 3 Vitalys Obesity Clinic, part of Rijnstate Hospital, Arnhem, Netherlands; 4 Divison of Human Nutrition and Health, Wageningen University, Wageningen, Netherlands; 5 Department of Intellectual Property & Standards, Philips, Eindhoven, Netherlands; 6 Clinical Research Center, Rijnstate Hospital, Arnhem, Netherlands

## Abstract

**Introduction:**

Remote assessment of patients after same-day discharge metabolic and bariatric surgery (MBS) might be improved by continuous remote monitoring (CRM), because it provides heart and respiratory rate (HR, RR), alongside patient complaints. This study aimed to evaluate the added value of CRM on clinical decision making in the remote assessment of patients after same-day discharge MBS.

**Methods:**

A nationwide survey among MBS healthcare professionals (*n* = 23) evaluated the clinical applicability of remote monitoring with general questions and its influence on clinical decision making using fictitious patient cases. Additionally, in a retrospective cohort study (*n* = 213), current remote patient assessment, HR and RR, technical sensor performance and patient satisfaction in (non-)readmitted patients who underwent same-day discharge MBS was evaluated.

**Results:**

The survey showed that HR and RR along with complaints does not lead to uniform treatment policies, and only 54% of healthcare professionals always use CRM vital signs in patient assessment. The cohort study shows that CRM data was used in only 20% of remote patient assessments, however when CRM data was used, all patients with normal data but with complaints were still referred to the emergency department. Additionally, no statistically significant differences were found in HR and RR between readmitted (*n *= 14) and non-readmitted patients (*n *= 199), 49% of HR and 43% of RR data were missing and 72% of patients would have undergone same-day discharge MBS without CRM.

**Conclusion:**

For this specific low-risk population, CRM did not appear to influence clinical decision making after same-day discharge MBS, under the current implementation conditions.

**Supplementary Information:**

The online version contains supplementary material available at 10.1007/s11695-026-08729-8.

## Key Points

HR and RR along with complaints does not lead to uniform treatment policies

CRM data is barely used in daily practice for remote patient assessment

After MBS complaints seem to outweigh vital signs in remote patient assessment

CRM in a low-risk population does not add value in same-day discharge MBS

**Graphical abstract:**

**Supplementary Information:**

The online version contains supplementary material available at 10.1007/s11695-026-08729-8.

## Graphical abstract

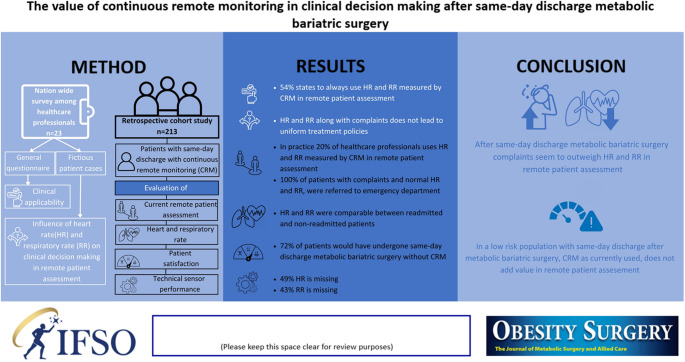

## Introduction

In 2022, 890 million adults worldwide suffered from obesity [1]. This has led to nearly 600,000 bariatric surgeries and endoscopic procedures globally in 2021 [2]. Due to high care demand, shortages of medical personnel, and growing numbers of chronically ill patients, accommodating this high volume of bariatric procedures is challenging [3].

Metabolic and Bariatric Surgery (MBS) with same-day discharge could contribute to increasing efficiency in the use of hospital capacity. Based on current literature, over 50,000 MBS with same-day discharge have already been performed globally. According to this, same-day discharge appears to be safe, with high success rates (88–99%), but with varying 30-day emergency department (ED) visits (0.1–10%) and hospital readmission rates (0.6–20%), which consequently could jeopardize hospital capacity [3–5]. Therefore, the variation in ED visits and hospital readmission rates in the aforementioned studies warrant further research. Some of the ED visits and hospital readmissions might have been prevented if, during the remote patient assessment at home, the patients’ vital signs would have been available along with their complaints.

Wearable sensors make it possible to monitor vital signs continuously, hence facilitating continuous remote monitoring (CRM) in hospital or even from a distance at home. Research shows that in-hospital CRM can identify abnormal vital signs that nurses in hospital wards would detect belatedly or miss completely [6]. Furthermore, it has the potential to recognize irregular patterns in heart rate (HR) and respiratory rate (RR) of surgical patients developing complications, and contribute to earlier detection of complications in patients after MBS [7]. However, for CRM to have additional value in daily clinical practice, it must have good clinical applicability (i.e. easy to use), and the ability to improve clinical decision making. To improve clinical decision making, abnormal vital sign patterns should indeed be present in patients who should be readmitted. Other important factors to assess the added value of CRM are technical sensor performance and patient satisfaction. To our knowledge, the added value of CRM on clinical decision making in the remote assessment of patients after same-day discharge MBS has not been evaluated yet.

Therefore, the first part of this study, a survey amongst MBS healthcare professionals, aims to assess the added value of remote monitoring in same-day discharge MBS. This will be done by evaluating its clinical applicability and the influence of vital signs in clinical decision making in the remote assessment of patients. Additionally, the second part of this study, a retrospective cohort study, aims to evaluate the current remote patient assessment, HR and RR data, technical sensor performance, and patient satisfaction.

## Methods

### Study Design

This study consists of a nationwide survey among MBS healthcare professionals and a retrospective cohort study in a center using CRM in same-day discharge. This study was approved by the local ethical committee of Rijnstate hospital (study number: 2023-2317). 

### Nationwide Survey amongst MBS Healthcare Professionals

To determine the clinical applicability and influence of remote monitoring of HR and RR on clinical decision making in remote patient assessment, we constructed a survey for healthcare professionals that was pilot-tested by a bariatric surgeon. This survey was shared with all bariatric centers in the Netherlands (*n* = 20) at a conference, and in a WhatsApp group. The survey consisted of a general questionnaire about clinical applicability (e.g. whether remote monitoring was available in that center and if it was used) and three fictitious patient cases to evaluate the influence of HR and RR on clinical decision making. The complete survey can be found in Supplementary Material 1. In case 1 the patient had no postoperative complaints, in case 2 the patient experienced postoperative abdominal pain with minimal nausea. Case 3 consisted of 2 parts, in the first part the patient experienced postoperative nausea and in the second part the same patient deteriorates with frequent vomiting. The healthcare professionals were asked in multiple choice questions what their treatment policy would be: (A) Expectant, instruct the patient to call in case of deterioration, (B) Medication (pain medication in case 2, antiemetics in case 3 part 1), (C) Refer to ED, (D) Other, with an explanatory note. The treatment policy was asked for each of the following HR and RR compositions: a: no HR and RR given, b: normal HR and RR, c: abnormal HR or RR, d: abnormal HR and RR. A HR between 50 and 100 bpm and a RR between 8 and 20 bpm were considered as normal. The values for HR and RR used in the cases were based upon expert opinion (bariatric surgeon and surgical resident) and what they thought to be a clinically relevant abnormal value for heart and/or respiratory rate. Note that in case 1 and case 3 part 2: the option “medication” was not given, because it was not applicable.

### Retrospective Cohort Study in a Large Teaching Hospital using CRM

A large teaching hospital (Rijnstate, Arnhem, the Netherlands) started with same-day discharge MBS with CRM in July of 2022. All patients until December 2023 that underwent same-day discharge MBS with CRM were included. Patients at low-risk for readmission and complications were eligible for same-day discharge, criteria for same-day discharge MBS can be found in Supplementary Material 1.

### Continuous Remote Monitoring Procedure

A wireless wearable sensor, the Healthdot (Philips Electronics B.V., the Netherlands), was used for CRM. The sensor was placed at the patient’s chest before discharge, and continuously measured HR, RR, posture, and activity level. Measurements of HR and RR by this sensor were validated in a bariatric population [8]. Activity level was measured on a scale of 0 (= minimum activity) to 10 (= maximum activity). In this study we did not analyze posture data. The measures were averaged every five minutes and transmitted to a cloud server via a low-power wide-area network. The Virtual Care Centre (VCC), an in-hospital ward staffed with nurses, monitored patients’ vital signs and symptoms daily between 7.15 AM and 11 PM [9]. These nurses could access the patients’ vital signs via a dashboard (IntelliVue Guardian Software version E.01.00). A notification was sent to the dashboard if three consecutive measurements were abnormal (HR: <50 or > 100 beats per minute (bpm), RR: <5 or > 20 breaths per minute (bpm)), after which a VCC nurse called the patient to evaluate their wellbeing, and if necessary, referred the patient to the ED. During the night, monitoring by the VCC did not take place. In the following morning the VCC nurses reviewed the previous night’s HR and RR and called patients who had ≥ 2 notifications. If patients experienced postoperative complaints, they had to call the VCC during the day or the nursing ward during the night. As part of standard care, a telephone consultation with a VCC nurse followed during the first postoperative day to evaluate the wellbeing of the patient.

Patients who underwent MBS before August 2023 wore the sensor for 72 h. From September 2023 onwards, patients wore the sensor until the telephone consultation on the first postoperative day, which took place around 3 PM.

### Data Collection

The following data were collected from the electronic medical record: patients characteristics including: age, gender, body weight, body height, BMI, obesity related comorbidities (gastroesophageal reflux disease, hypertension, diabetes mellitus type 2, dyslipidemia) and smoking status; surgery details including: type of surgery and complications during surgery; post-operative course including: 30-day ED visits, hospital readmissions and complications; number and reason for extra contact moments with VCC. The CRM (averaged 5 min) measurements on HR, RR and physical activity level along with timestamp data were collected from the start of the monitoring until 11:00 AM the next day (regular discharge time of inpatient MBS), or until one hour before readmission, in case of readmission before 11:00 AM. The last hour before readmission was excluded because it includes transfer to the hospital, which may affect HR and RR. Additionally, the number of sensor replacements per patient was collected.

### Remote Patient Assessment

The remote assessment of patients prior to ED visit or hospital readmission was evaluated, by describing if and how HR and RR from CRM was used in clinical decision making, based on reports from the medical record.

### Analysis of HR and RR in (non) Readmitted Patients

Median values of average HR, RR and activity level of individual patients were compared between patients with and without re-admission, to observe potential differences that could indicate deterioration. In addition, the median HR and RR in readmitted patients were compared between the second last hour before readmission (T1) and the period from start of monitoring until 2 h before readmission (T0, see Fig. 4b).

### Technical Sensor Performance

The technical performance of the sensor was evaluated by describing the number of sensor replacements per patient, mean measurement duration, missing vital sign data concerning: total intended measurements, number of missing measurements, percentage of missing measurements per patient, and the number of missing periods of ≥ 1 h per patient (i.e., no measurements for a consecutive period of ≥ 1 h; because hourly measurements were considered clinically relevant), and the reason for missing values retrieved from the electronic medical record.

### Patient Satisfaction

To evaluate patient satisfaction with same-day discharge MBS, patients completed an online postoperative multiple-choice questionnaire as part of standard care. These include: (1) Did you feel safe at home?, (2) Did the sensor have added value for you?, (3) Would you have had same-day discharge surgery without the sensor? (4) How did you feel about same-day discharge before surgery?, (5) How did you feel about same-day discharge after surgery? The answers were retrieved from OnlinePROMS (version 2024.9.5, Interactive Studios).

### Statistical Analysis

Continuous data were expressed as mean (standard deviation (SD)) or in case of a non-normal distribution as median [25th percentile(Q1)-75th percentile(Q3], categorical data were expressed as number with percentage. Descriptive statistics (medians, percentages and counts) were used to describe the monitoring period and (periods) of missing measurements.

To compare patient characteristics and vital sign data between the group with and without hospital readmission the Mann-Whitney U (continuous data) was used, because of a non-normal distribution. For categorical data the Fisher’s exact (proportions) test was used. A p-value < 0.05 was considered statistically significant.

Analyses were done using SPSS Statistics (v.29.1, IBM, New York, United States), except vital sign data which was analyzed using R version 4.3.0 (R foundation, 2020, Vienna, Austria).

## Results

### Nationwide Survey amongst MBS Healthcare Professionals

A total of 23 healthcare professionals working in 8 (out of 20) Dutch bariatric centers filled out the general part of the survey, of whom 12 were surgeons, 9 were surgical residents, 1 was a PhD candidate and 1 was a nursing specialist in training.

### Clinical Applicability

**Fig. 1:**
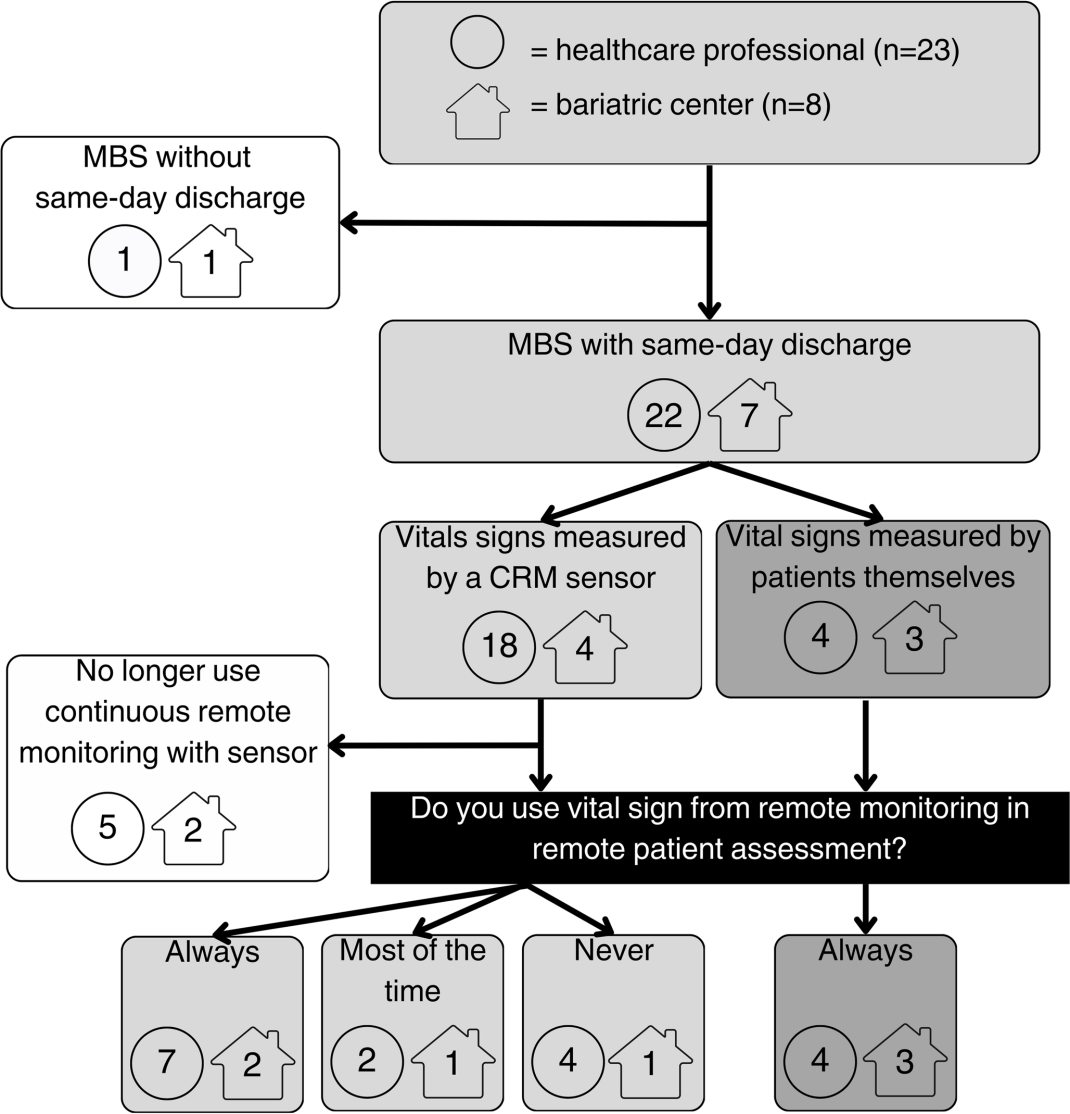
Flowchart of same-day discharge MBS and the use of remote monitoring from the nationwide survey among 23 MBS healthcare professionals from 8 bariatric centers

Out of the 8 bariatric centers, 7 (87.5%) offered same-day discharge MBS, of which 2 no longer used CRM. For additional details see the flowchart in Fig. 1. The reasons for the 2 bariatric centers to stop using CRM was because physicians believed that CRM had no added value, complications were not detected or because of high costs. In bariatric centers where patients measured vital signs themselves (e.g., with a pulse oximeter), vital signs were always used in the remote assessment of patients at home. In the 4 bariatric centers where a sensor was used to monitor patients at home, 7 (54%) healthcare professionals always use, and 4 (31%) never use vital signs in the remote assessment. Reasons for not using the vital signs were not knowing where to access and view the data (*n* = 3, from 1 center) and the opinion that complaints are more important than vital signs (*n* = 1).

### Influence of Vital Signs on Clinical Decision Making

**Fig. 2:**
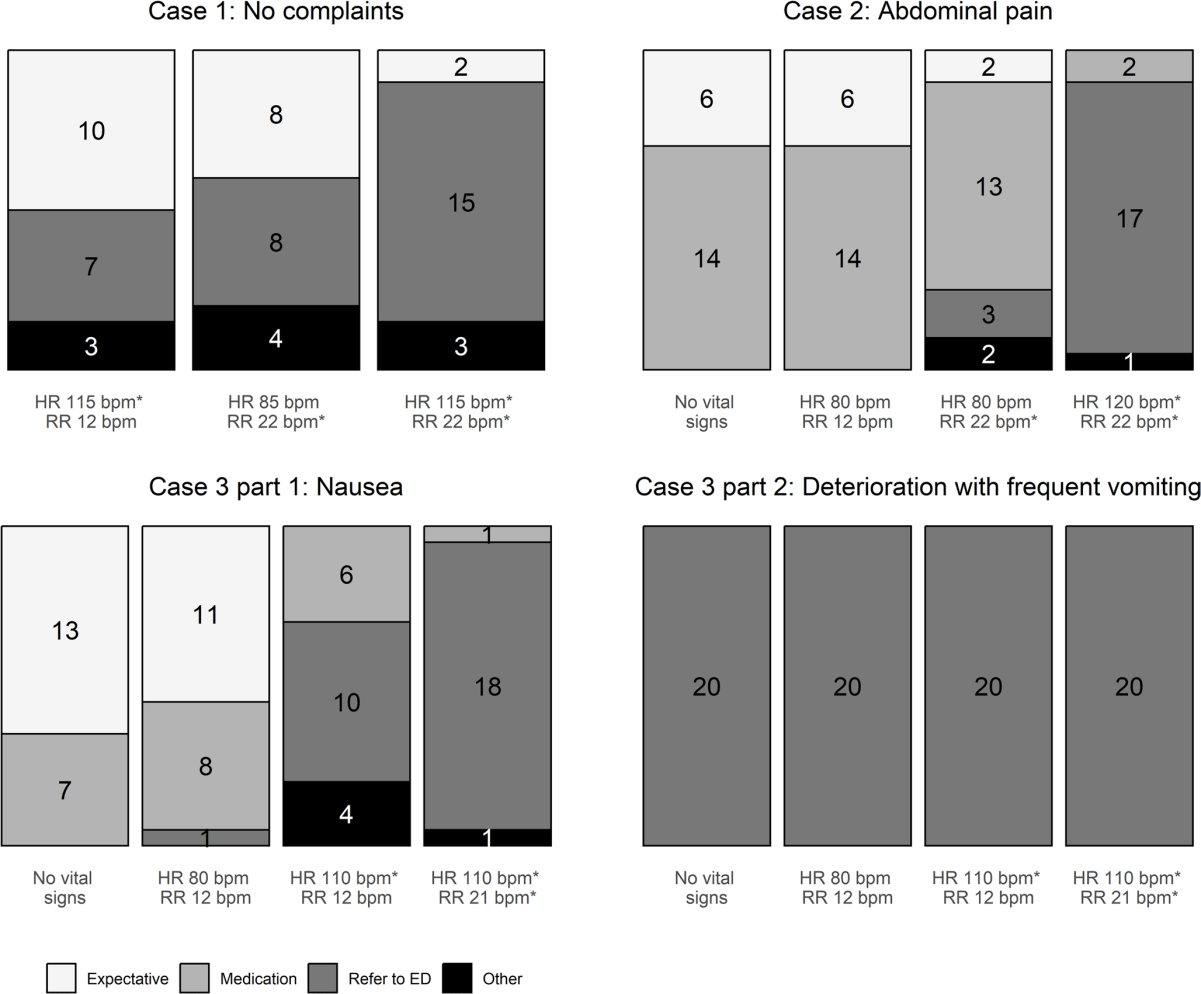
Results of 3 fictitious patient cases to evaluate the role of heart rate and respiratory rate on clinical decision making. Bpm: beats/breaths per minute, ED: Emergency Department, HR: Heart Rate, RR: Respiratory Rate. *abnormal rate for HR or RR

The part of the survey concerning fictitious patient cases was filled out by 20 healthcare professionals. The results can be found in Fig. 2. In general, patients were not sent to the ED if HR and RR were absent or normal, and were sent to the ED if both the HR and the RR were abnormal. An exception was case 3 part 2, in which the patient deteriorates and had complaints of frequent vomiting. In this case, the complaints appeared to be severe enough for the patient to be sent to the ED, regardless of the vital signs. The answer “other” was chosen 18 times and generally referred to further exploration of the complaints by telephone or telephonic reassessment within a few hours.

### Retrospective Cohort Study in a Large Teaching Hospital using CRM

In total, 216 patients underwent same-day discharge MBS with CRM of which 3 (1%) patients were excluded for vital sign data comparison because no sensor data was received. The median age was 39.0 [30.0–49.5] years, BMI of 42.0 [39.3–45.3] kg/m^2^, and 167 (78%) underwent laparoscopic Roux-and-Y Gastric Bypass (Table 1). Within 30-days after surgery, 14 (7%) patients were readmitted, of which one patient was readmitted twice, resulting in a total of 15 hospital readmissions (Table 2). The majority of the patients (*n* = 11; 73%) was readmitted on the first postoperative day. Patients were readmitted because of hematemesis or melena (*n* = 6; 40%), nausea and vomiting (*n* = 4; 27%), and insufficient oral intake (*n* = 4; 27%). Patients were mainly treated conservatively (*n* = 13; 87%), e.g. antiemetics, proton pump inhibitors and clinical observation. One patient was readmitted due to postoperative bleeding requiring surgical intervention. Patient characteristics were comparable between the 14 readmitted and the 199 non-readmitted patients. Readmission details per patient can be found in supplementary Table 1.

**Table 1:** Patient characteristics for the total study population (*n* = 213) and stratified for patients without (*n *= 199) and with hospital readmission (*n *= 14)

Characteristic	All(*n *= 213)	Without readmission (*n* = 199)	With readmission (*n* = 14)	*p*
Gender (female)	174 (81.7)	162 (81.4)	12 (85.7)	.51
Age (years)	39.0 [30.0-49.5]	39.0 [30.0-49.0]	36.5 [30.8-51.5]	.95
Body Mass Index (kg/m^2^)	42.0 [39.3-45.3]	42.0 [39.3-45.5]	41.3 [38.5-43.2]	.29
Obesity related comorbidities				
GERD	35 (16.4)	31 (15.6)	4 (28.6)	.18
Hypertension	34 (16.0)	33 (16.6)	1 (7.1)	.31
DM type 2	17 (8.0)	17 (8.5)	0 (0.0)	.30
OSA	15 (7.0)	14 (7.0)	1 (7.1)	.65
Dyslipidemia	14 (6.6)	14 (7.0)	0 (0.0)	.37
Smoking				1.0
Non-smoker	91 (42.7)	85 (42.7)	6 (42.9)	
Smoker	29 (13.6)	27 (13.6)	2 (14.3)	
Former smoker	93 (43.7)	87 (43.7)	6 (42.9)	
Type of surgery				.56
LRYGB	167 (78.4)	157 (78.9)	10 (71.4)	
LSG	44 (20.7)	40 (20.1)	4 (28.6)	
Minimizer	2 (0.9)	2 (1.0)	0 (0.0)	

Data are presented as median [Q1-Q3] or number (percentage), *GERD* gastroesophageal reflux disease, *DM* diabetes mellitus, *OSA* obstructive sleep apnea, *LRYGB* laparoscopic Roux-and Y gastric bypass, *LSG* laparoscopic sleeve gastrectomy

**Table 2:** Details of 15 hospital readmissions in 14 patients

	n (%)
Reason readmission	
Hematemesis and or melena	6 (40.0)
Nausea and vomiting	4 (26.7)
Insufficient intake	4 (26.7)
Postoperative bleeding	1 (6.7)
Day readmission	
PO day 0	1 (6.7)
PO day 1	11 (73.3)
PO day 2	1 (6.7)
PO day 4	1 (6.7)
PO day 7	1 (6.7)
Vital signs sensor used in pre-hospital evaluation prior to readmission	
No	10 (66.7)
Yes	3 (20.0)
Not applicable*	2 (13.3)
Treatment	
Conservative#	13 (86.6)
Re-operation	1 (6.7)
Blood transfusion	1 (6.7)

*because the monitoring period had ended, #use of proton pump inhibitors, anti-emetics, clinical observation, *PO* post-operative

### Remote Patient Assessment

In 12 out of 15 hospital readmissions the HR and RR was not used in the assessment prior to readmission. In 2 of these the monitoring period had ended, for the other 10 hospital readmissions the reason for not using the HR and RR was unknown. Of the patients for whom HR and RR were used prior to readmission (*n* = 3), two patients called with complaints to the nursing ward and were referred to the ED, despite having normal values for HR and RR. The third patient was called by the VCC nurse because of a high RR, which in combination with the patient’s postoperative complaints led to hospital readmission. The patient with a postoperative bleeding, contacted the VCC himself because of worsening complaints of nausea and vomiting. The patient then was referred to the ED. In the second last hour before readmission the RR was normal (17 bpm) and HR from CRM was not available for unknown reasons.

A total of 6 patients (2.8%) visited the ED but were not readmitted. Reasons for ED visits included abdominal pain (*n* = 3), vasovagal collapse (*n* = 1), obstructive ureteral stone (*n* = 1) and tingling and heavy feeling in left arm (*n* = 1). In the remote patient assessment HR and RR were used in 1 of these patients, which were normal, but based on the complaints the patient was still referred to the ED. In the other 5 patients, HR and RR were not available, because the monitoring period had already ended at the start of their complaints.

### Analysis of HR and RR in (non-)Readmitted patients

**Fig. 3:**
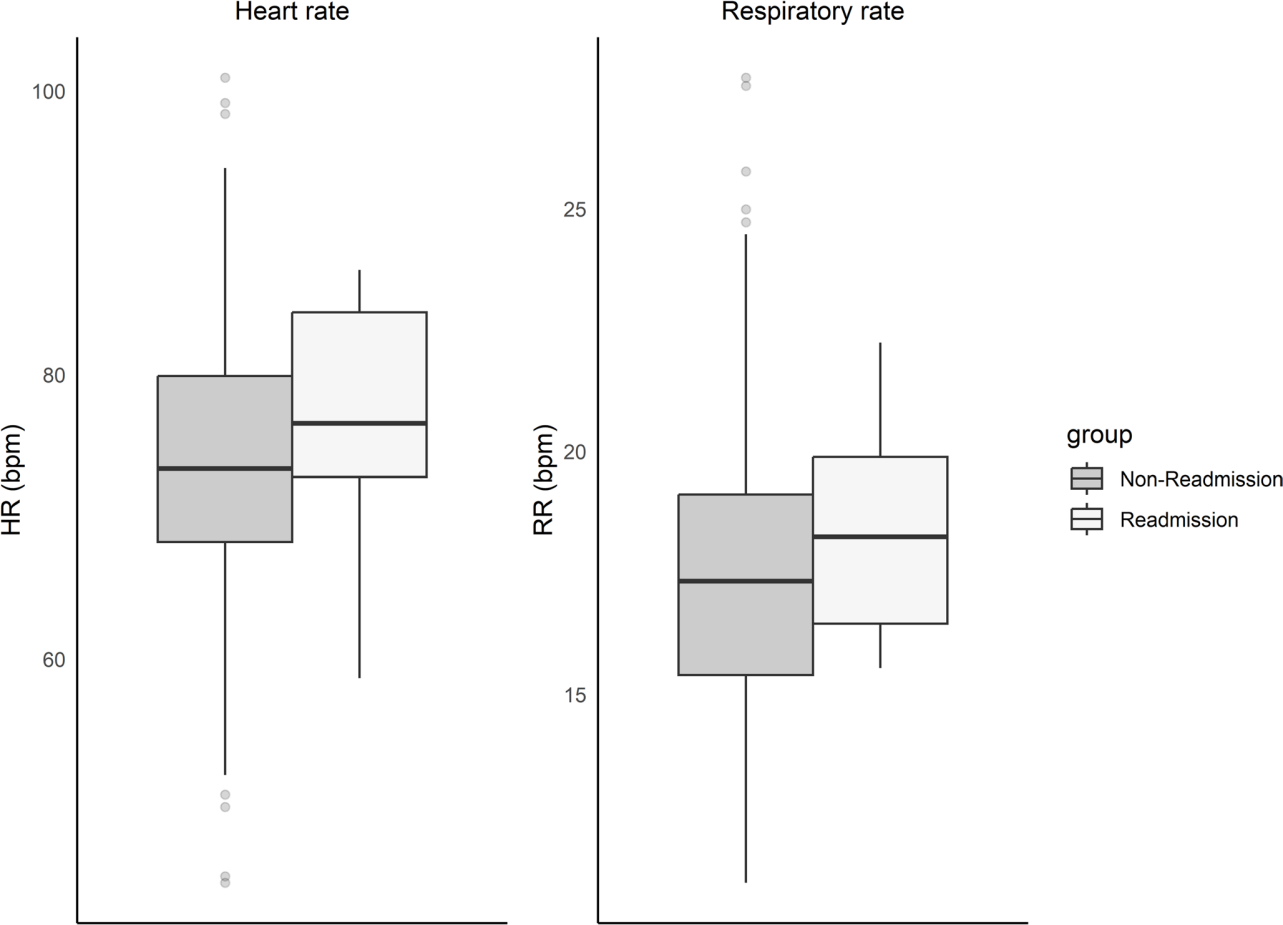
Average heart rate and respiratory rate of readmitted patients (*n* = 14) versus non-readmitted patients (*n* = 199) bpm: beats/ breaths per minute, HR: heart rate, RR: respiratory rate

Sensor data from readmitted (*n* = 14) and non-readmitted patients (*n* = 199) had an average HR of 76.6 bpm [72.8–84.4] vs. 73.4 bpm [68.2–79.9] (*p*=.16), and average RR of 18.3 bpm [16.5–19.9] vs. 17.3 bpm [15.4–19.1] (*p*=.12) (Fig. 3). Average activity levels were 4.0 [3.3–4.2] vs. 3.3 [3.0-3.8] (*p*=.05). Limiting the analysis to patients with > 50% data coverage resulted into similar findings (supplementary Table 2).

**Fig. 4:**
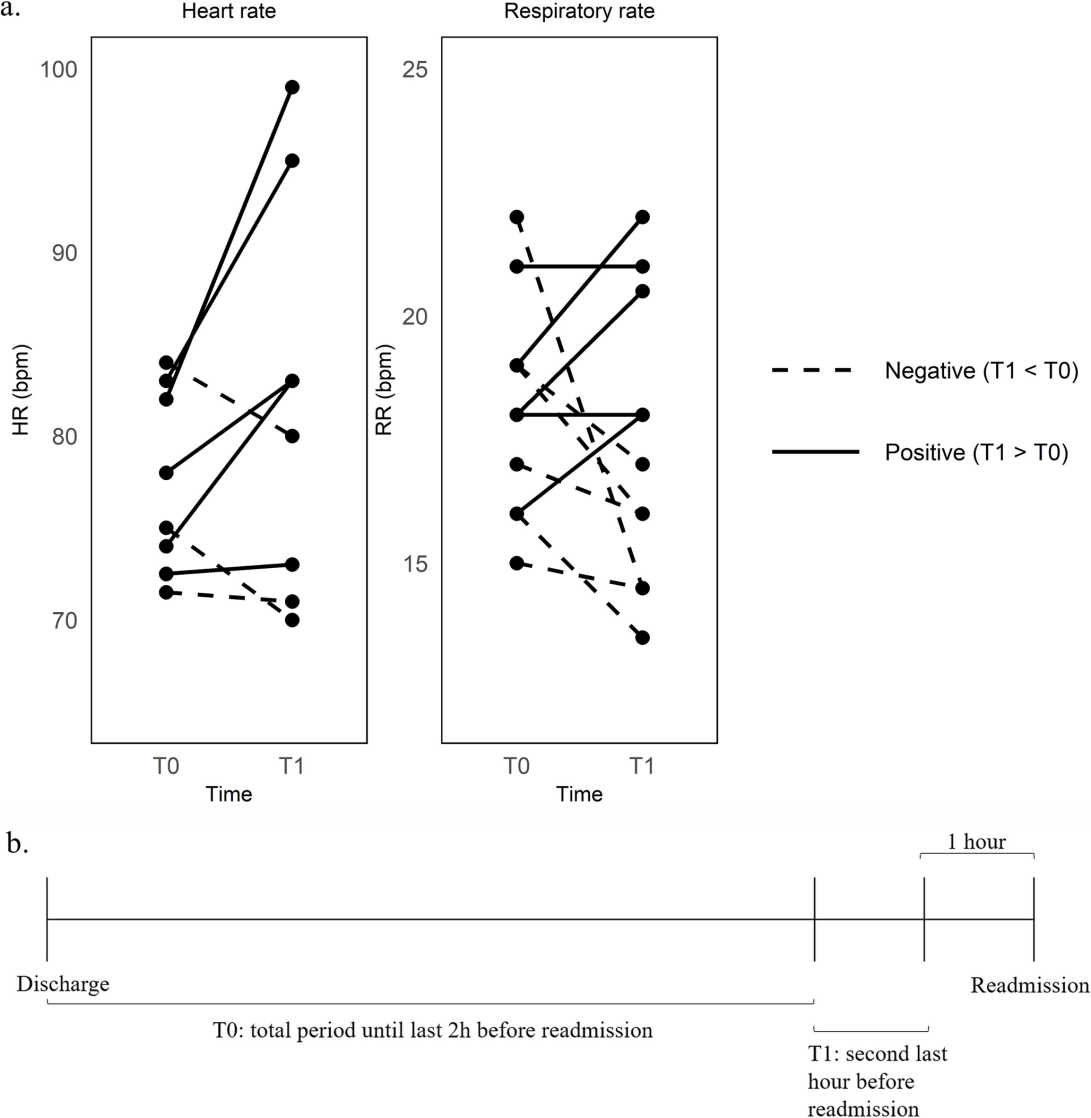
(**a**) Median heart (*n* = 8) and respiratory rate (*n* = 11) in T0 and T1 of readmitted patients (**b**) Timeline showing the definition of T0 and T1 from discharge to hospital readmission. T0: the period from start of monitoring until 2 h before readmission, T1: second last hour before readmission, HR: Heart Rate, RR: Respiratory Rate, bpm: beats/breaths per minute

In the analysis of 14 readmitted patients, HR in the second-last hour before readmission (T1) was available for 8 patients (57%) and RR for 11 patients (79%). For HR, the majority (5 out of 8 patients), had a relatively higher HR in the second last hour before readmission (T1) compared to the total previous monitoring period (T0). For RR this was seen in 5 out of 11 patients (Table 3; Fig. 4). Supplementary Fig. 1 shows the patterns of HR and RR of the patients with > 50% data coverage until readmission.

**Table 3:** Median heart and respiratory rate in T0 and T1 of readmitted patients

Patient	T0		T1	
	HR	RR	HR	RR
1	71.5	18	71	20.5
2	78	19	83	16
3	64	22	-	14.5
4	72.5	21	73	21
5	97	19	-	17
6	74	18	83	18
7	84	16	80	18
8	82	17	99	16
9	74	16	-	13.5
10	75	15	70	14.5
11	83	19	95	22

T0: the period from start of monitoring until 2 h before readmission, T1: second last hour before readmission, HR: Heart Rate, RR: Respiratory Rate, bpm: beats/breaths per minute

### Technical Sensor Performance

**Fig. 5:**
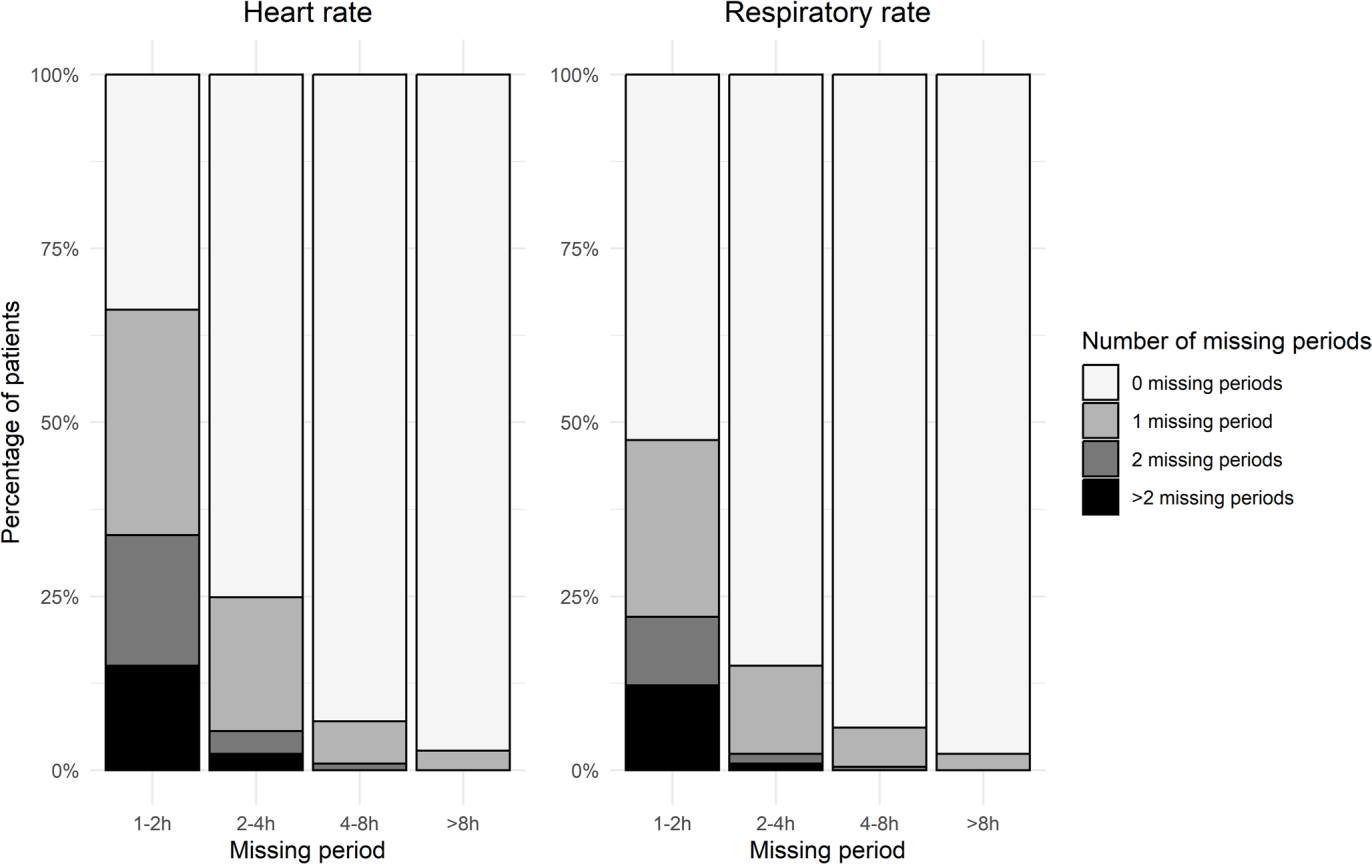
Percentage of patients (*n* = 213) with the duration and number of missing periods (missing consecutive measurements ≥ 1 h) of heart rate and respiratory rate

In-hospital replacement of the sensor was needed in 24 out of 213 patients, of which 4 patients needed a second replacement because the sensor was not functioning. The mean duration of the monitoring was 75.9 h [39.4–79.8]. All patients had missing measurements for both HR and RR. The percentage of missing measurements across the entire monitoring period was 49.0% for HR and 42.7% for RR, this was comparable to the percentage of missing measurements per patient with HR 50.2% [28.8–68.9] and RR 42.6% [22.8–64.4]. In 38.1% of the measurements all vital sign data was missing (HR, RR, activity level and posture), which indicates connection issues. Possible reasons for these connection issues that were mentioned were: living in newly built houses, body position and blockage of the sensor e.g. with a pillow. In the majority the possible reasons for connection issues were not described. The duration of a missing period (no measurements for a consecutive period of ≥1 h) had a median of 1.5 h [1.2–2.1] for HR, and 1.5 h [1.2-2.0] for RR. The number, duration and percentage of missing periods can be seen in Fig. 5. For example, the first stacked bar of HR shows that HR is missing for 1–2 h in 62% of the patients, of which 32% had one missing period of 1–2 h, 19% had two and 15% had more than two missing periods.

Apart from telephonic consultations as part of standard care, the VCC nurses contacted 51 patients (24%) because of connection issues. This resulted in a total of 65 additional phone calls to these 51 patients to check their wellbeing. None of these patients were readmitted or experienced severe symptoms during this non-receiving period.

### Patient Satisfaction

The questionnaire was sent to all patients (*n* = 213), questions 1–3 were filled in by 142 patients and questions 4 and 5 by 149 patients, of whom 8 were readmitted.

In retrospect, both non-readmitted and readmitted patients felt positive about same-day discharge before surgery, after surgery this percentage increased for non-readmitted patients but decreased in readmitted patients.

**Fig. 6:**
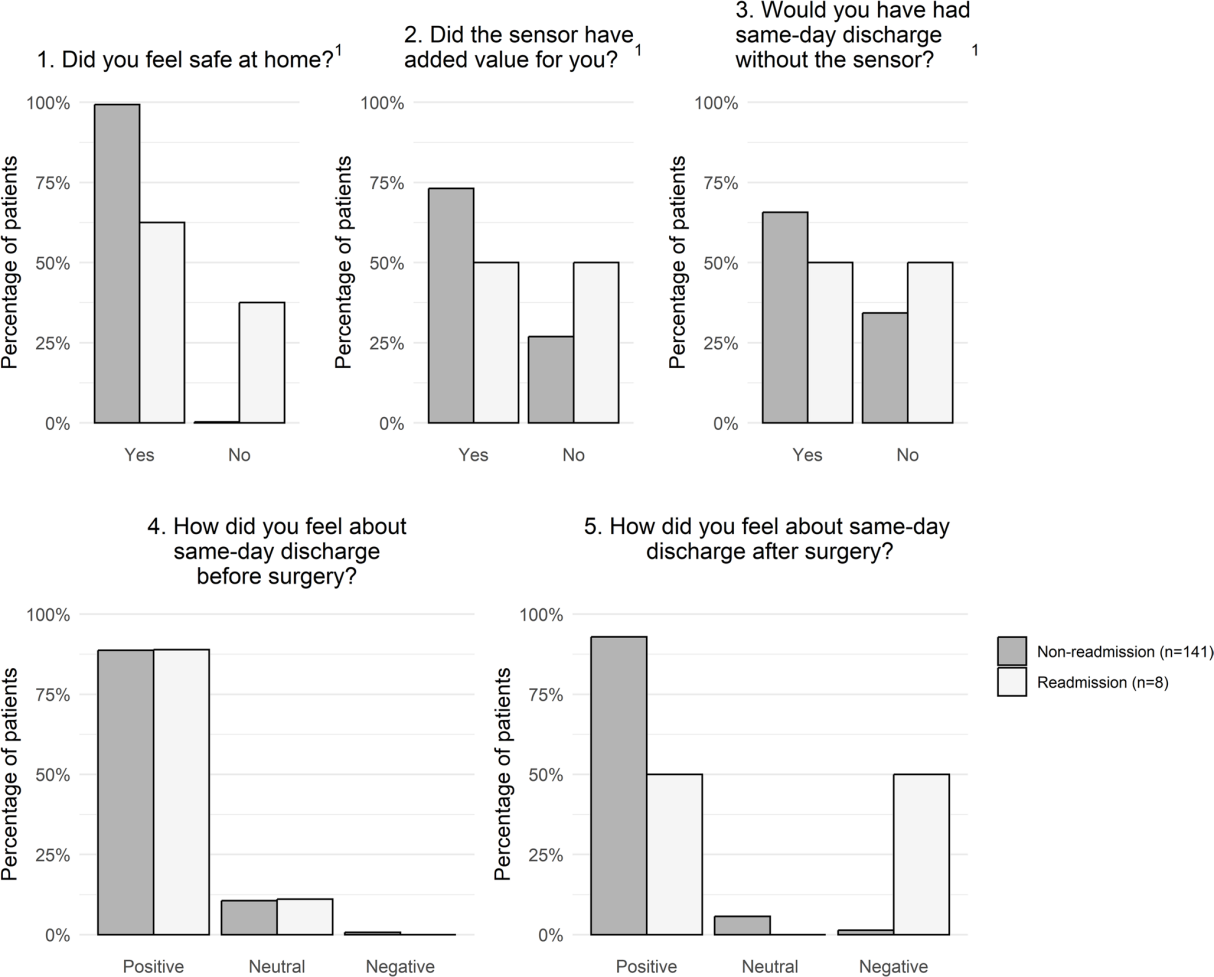
Questionnaire on patient satisfaction of same-day discharge metabolic bariatric surgery with continuous remote monitoring. ^1^Non-readmission group *n* = 134

Significantly more non-readmitted patients felt safe at home compared to readmitted patients (99.3% vs. 62.5%, *p*<.001), see Fig. 6. A majority reported that the sensor had added value for them. However, in retrospect, a majority of the patients would have undergone same-day discharge MBS without a sensor.

## Discussion

The survey shows that only 54% of the healthcare professionals claim to always use vital sign data measured by the sensor. Additionally, the various answers in the fictitious patient cases of the survey show that addition of HR and RR alongside patient’s complaints does not lead to uniform treatment policies, which makes the added value in clinical decision making questionable. The retrospective cohort study shows little actual use of sensor data in daily practice for remote patient assessment, and when sensor data was used, the majority was still referred to the ED despite having normal HR and RR. This indicates that after MBS complaints outweigh vital signs in remote patient assessment. Additionally, in the analysis of HR and RR no differences between readmitted and non-readmitted patients were found. Furthermore, technical sensor performance was quite poor in terms of missing periods, and the majority of patients reported that they would have also undergone same-day discharge MBS without a sensor. Overall, this does not support the use of CRM with a sensor in the remote patient assessment in same-day discharge MBS in this low-risk population.

The survey shows that in bariatric centers where patients themselves measure vital signs, all healthcare professionals state that they always use vital signs in remote assessment of these patients, compared to half of healthcare professionals that use a sensor for CRM. In practice, revealed by our retrospective cohort study, it even appears that vital signs measured by the sensor are only used in 20% of remote patient assessment prior to hospital readmission. This shows that the measurements of vital signs by patients themselves benefits clinical applicability, probably because these vital signs are easily accessible by asking the patient during telephonic assessment of their complaints. The majority of healthcare professionals that stated that the reason they did not use sensor vital signs was because of lacking awareness where to access these measurements. This is consistent with a review indicating that successful CRM implementation requires investment in infrastructure, integration with electronic medical records for ease of use and interpretation, and workflow integration [10]. Increased awareness and easy accessibility of CRM vital sign data for healthcare professionals would likely increase the use in remote patient assessment.

However, even if CRM vital sign data is accessible, the fictitious patient cases in our survey showed various treatment policies among healthcare professionals, with the exception of frequent vomiting where the patient was referred to the ED, independent of the vital signs. This might indicate that, in patients after MBS, postoperative complaints outweigh the value of vital signs. This is consistent with the results of our retrospective cohort study, in which all patients with normal vital signs (*n* = 3) during remote assessment were still referred to the ED because of their complaints. While complaints outweigh vital signs it must be noted that the CRM data provided in the fictitious patient cases shows single-value vital signs, potentially limiting clinical decision-making as trends could provide more insight into the patient’s status. If healthcare professionals were accustomed to the working method and more vital sign data was available, CRM with trend data could perhaps offer added value.

With regard to sensor data, no statistically significant differences were found in the average HR and RR between readmitted and non-readmitted patients. However, in the majority of readmitted patients (63%) HR increased in the second last hour before hospital readmission. These results broadly agree with previous research on CRM in high-risk surgery patients, showing that both HR and RR increase in the hours before an adverse event [11]. It should be noted that in our population, while an increase in HR was seen, it remained below 100 bpm in readmitted patients, and only 45% of the patients had an increase in RR. This is in line with the reasons for hospital readmission, as the majority (53%) was readmitted because of nausea, vomiting and insufficient intake. With these complaints, there are reasons for observation, but one would not expect to see large changes in HR or RR as would be seen with postoperative bleeding or anastomotic or staple-line leakage [11]. Even in such severe complications, early postoperative elevations in HR may fall within a clinically acceptable range, which may limit their diagnostic value.

These results only apply to low-risk patients. Higher risk patients might benefit more from CRM. Perhaps CRM could be targeted to specific patients in the future rather than applied universally. In our study, only one patient was readmitted with a postoperative bleeding. Unfortunately, HR measurements were not available in the second last hour before hospital readmission in this patient. This patient had missing HR data at a critical time point, showing that consistent HR and RR availability of the sensor is essential.

Unfortunately, the overall percentage of missing data was high in our study, with 49% of HR and 43% RR missing, limiting the ability to detect clinically meaningful trends. The retrospective design of the cohort study relies on the availability of existing data. The amount of missing data is higher than a comparable study using the same sensor with < 25% for HR and < 10% of RR data missing on the first postoperative day [12]. Methodological differences, e.g. measurement frequency and use of overall percentage versus median values, may explain these differences.

With regard to patient satisfaction, the majority of the patients reported that the CRM sensor had added value for them. However, a majority of the patients would also have undergone same-day discharge MBS without CRM (64.8%). These varying answers make it questionable if CRM with a sensor really adds to patients’ satisfaction, or whether the sensor mainly offers an additional but not strictly necessary feeling of safety. Future research could explore patients’ underlying reasoning in more depth to better understand how they assess the necessity and value of CRM.

A major strength of this study is its comprehensive analysis, taking into account many factors, like clinical applicability, patient satisfaction, and real-world implementation data providing a complete overview for the value of CRM in same-day discharge MBS. Another strength is that the survey was conducted nationwide, resulting in an overview of 8 different bariatric centers, including different types of remote monitoring and healthcare professionals with different roles and thus reflecting daily practice. Additionally, reporting the observed technical failures and openly discussing implementation challenges contribute to a more realistic and methodologically robust interpretation of our findings.

However, there are some limitations that must be acknowledged as well. The survey was carried out in 8 out of 20 bariatric centers in the Netherlands, with different same-day discharge protocols and remote monitoring, whereas the retrospective cohort study was carried out in a single bariatric center that uses CRM with a sensor. Our fictitious case study included only 20 participants. Furthermore, our study included 213 patients. Even though this is one of the largest studies of its kind, this may still be considered a rather limited number of patients, and the study may therefore be underpowered to detect meaningful differences in the retrospective analysis. Implementation shortcomings such as lack of provider engagement, data inaccessibility and the large amount of missing data likely contributed to the findings that CRM lacks value. The monitoring duration was initially 72 h but was shortened to postoperative day 1 after the initial implementation phase. This change is unlikely to have affected the results, as most readmissions occurred within 24 h of discharge.

In conclusion, our study results apply to patients who are initially already considered to be at low-risk for readmission and complications as they are discharged the same day after surgery. In this specific group, CRM did not appear to influence clinical decision-making regarding same-day discharge MBS under the current implementation conditions, characterized by low CRM usage and substantial missing sensor data. Patients were still referred to the ED despite having normal HR and RR values, and complaints seemed to outweigh vital signs in remote patient assessment in both the survey and retrospective analysis. No statistically significant differences were found in vital signs patterns between readmitted and non-readmitted patients, and the majority of patients reported they would have agreed to same-day discharge MBS without a sensor. However, due to the high percentage of missing data, no conclusions can be drawn on predictive value of vital sign data. Given the current implementation and technology issues, vital sign monitoring might still be beneficial once these issues are resolved. Future research is needed to determine whether CRM, perhaps with the application of artificial intelligence, has added value in the remote assessment of other patient populations.

## Supplementary Information

Below is the link to the electronic supplementary material.

Supplementary Material 1

## Data Availability

The data that support the findings of this study are available from the corresponding author upon reasonable request.
