# Supplementary material for: The value of Continuous Remote Monitoring in Clinical Decision Making After Same-day Discharge Metabolic Bariatric Surgery
Source: Obes Surg. 2026 Jun 11;36(7):3521–34. doi: 10.1007/s11695-026-08729-8 (PMC13323212; doi:10.1007/s11695-026-08729-8)
Supplement: Supplementary file 1 — Supplementary Material 1 [file 11695_2026_8729_MOESM1_ESM.docx]

**Supplementary materials**

Supplementary table 1: Details on readmission per patient

| No. | Reason readmission | Treatment | Postoperative day at readmission | Vitals signs senor used |
| --- | --- | --- | --- | --- |
| 1 | Nauseau and vomitting | Antiemetics | 1 | Yes |
| 2 | Nauseau and vomitting | Antimetic, iv fluids | 1 | No |
| 3 | Nauseau and vomitting | PPI | 4 | Not applicable |
| 4 | Nauseau and vomitting | Clinical observation | 1 | No |
| 5 | Hematemesis | Tranexamic acid | 1 | No |
| 6 | Hematemesis | Tranexamic acid | 1 | No |
| 7 | Hematemesis | Tranexamic acid | 1 | No |
| 8 | Hematemesis | No oral inake, morphine, antiemetics | 0 | Yes |
| 9 | Problems with oral intake | Antiemetics | 1 | No |
| 10 | Problems with oral intake | Observation | 1 | No |
| 11 | Problems with oral intake | Iv fluids and observation | 1 | No |
| 12 | Problems with oral intake | Observation | 1 | Yes |
| 13 | Rebleeding | DLS with removal of hematoma | 2 | No |
| 14 | Melena | Tranexamic acid, bloodtranfusion | 7 | Not applicable |
| 15 | Hematemasis and melena | PPI perfusor, tranexamic acid | 1 | unknown |

Supplementary material 1: Survey for healthcare professionals on influence of vital signs of remote monitoring on clinical decision making.

**Part 1: General questionnaire**
1. Gender
2. Age
3. Which bariatric center do you work in?

4. What is your role?

A. Surgeon

B. Surgical fellow

C. Surgical resident

D. Other + explanatory note

5. How much work experience do you have in your current role?

6. Do you have metabolic bariatric surgery with same-day discharge at your center?

7. Do you use remote monitoring in same-day discharge?
 A. Yes, with a wearable sensor
 B. Yes, patients measure vital signs themselves
 C. Yes, other + explanatory note
 D. No
 E. No, not anymore but before we used a wearable sensor

8. If a patient calls you with postoperative complains, do you use the vital signs of remote monitoring?

A. Yes, always
 B. Yes, sometimes
 C. Yes, most of the time
 D. No + explanatory note

**Part 2: Fictious case part**

Three short fictious patient cases will follow. In these cases vital signs measured by a wearable sensor [blinded] are used. This sensor is worn by patients after discharge. It measures heart rate and respiratory rate, among other things.

**Casus 1:**

Female 45 years old, BMI 45 kg/m^2^Medical history: hypertension, diabetes mellitus type 2

Medication: Metformin, amlodipine.

Surgery: Roux-en-Y gastric bypass, without peroperative complications, with same-day discharge.

As part of the standard case a telephone consultation was made on postoperative day 1. The patient reports that she is doing well, has sufficient oral intake, pain is manageable, no nausea, no other complaints.

You will now receive the following vital signs from the wearable sensor:

*Heart rate 112 bpm, respiratory rate 12 bpm*

2a. What would your treatment policy be?
 A. Expectant, instruct the patient to call in case of deterioration

B. Refer to emergency department (ED) or outpatient clinic

C. Other + explanatory note

Instead of the previous vital signs, you will now receive the following vital signs from the wearable sensor:

*Heart rate 85 bpm, respiratory rate 22 bpm*

2b. What would your treatment policy be?
 A. Expectant, instruct the patient to call in case of deterioration

B. Refer to emergency department (ED) or outpatient clinic

C. Other + explanatory note

Instead of the previous vital signs, you will now receive the following vital signs from the wearable sensor:

*Heart rate 115 bpm, respiratory rate 22 bpm*

2c. What would your treatment policy be?
 A. Expectant, instruct the patient to call in case of deterioration

B. Refer to emergency department (ED) or outpatient clinic

C. Other + explanatory note

**Follow up case 1**As the patient had no complaints, an expectant policy was followed. Further postoperative recovery remained uneventful. [End of case]

**Case 2**

Female 37 years old, BMI 40 kg/m^2^,

Medical history: bipolar personality disorder

Medication: Lithium

Surgery: Roux-en-Y gastric bypass, without per operative complications, with same-day discharge.

Postoperative day 1: The patient calls with increasing abdominal pain, she takes 1000mg paracetamol 4 times a day. She has adequate oral intake, some nausea and no vomiting.

3 a. What would your treatment policy be?
 A. Expectant, instruct the patient to call in case of deterioration

B. Pain medication, instruct the patient to call in case of no effect or deterioration

C. Refer to emergency department (ED) or outpatient clinic

D. Other + explanatory note

You will now receive the following vital signs from the wearable sensor:

*Heart rate 80bpm, respiratory rate 12 bpm*

2b. What would your treatment policy be?
 A. Expectant, instruct the patient to call in case of deterioration

B. Pain medication, instruct the patient to call in case of no effect or deterioration

C. Refer to emergency department (ED) or outpatient clinic

D. Other + explanatory note

Instead of the previous vital signs, you will now receive the following vital signs from the wearable sensor: *Heart rate 120 bpm, respiratory rate 22 bpm*

1c. What would your treatment policy be?
 A. Expectant, instruct the patient to call in case of deterioration

B. Pain medication, instruct the patient to call in case of no effect or deterioration

C. Refer to emergency department (ED) or outpatient clinic

D. Other + explanatory note

**Follow up case 2**The patient was referred to the ED, an abdominal CT showed no abnormalities. The patient was readmitted for clinical observation and pain relief. The patient could be discharged after 2 days and further postoperative course remained uneventful.

**Case 3**

Male 33 years old, BMI 43 kg/m^2^, medical history: none

Medication: none

Surgery: laparoscopic sleeve gastrectomy (LSG) without peroperative complications, with same-day discharge.

LSG +1 day : During the regular telephone consultation with the nurse, the patient states that he is nauseous, his oral intake is sufficient, no vomiting, no abdominal pain.

1a. What would your treatment policy be?
 A. Expectant, instruct the patient to call in case of deterioration

B. Antiemetics

C. Refer to emergency department (ED) or outpatient clinic

D. Other + explanatory note

You will now receive the following vital signs from the wearable sensor:

*Heart rate 80 bpm, respiratory rate 12 bpm*

1b. What would your treatment policy be?
 A. Expectant, instruct the patient to call in case of deterioration

B. Antiemetics

C. Refer to emergency department (ED) or outpatient clinic

D. Other + explanatory note

Instead of the previous vital signs, you will now receive the following vital signs from the wearable sensor: *Heart rate 110 bpm, respiratory rate 12 bpm*

1c. What would your treatment policy be?
 A. Expectant, instruct the patient to call in case of deterioration

B. Antiemetics

C. Refer to emergency department (ED) or outpatient clinic

D. Other + explanatory note

Instead of the previous vital signs, you will now receive the following vital signs from the wearable sensor: *Heart rate 110 bpm, respiratory rate 21 bpm*

1d. What would your treatment policy be?
 A. Expectant, instruct the patient to call in case of deterioration

B. Antiemetics

C. Refer to emergency department (ED) or outpatient clinic

D. Other + explanatory note

**Follow up case 3**

Patient received antiemetics and was instructed to call in case of no effect or deterioration.

LSG +2 days: Patient calls, he has been vomiting since this morning, cannot keep anything down, not even water or medication. No pain, no defecation yet, normal miction.


1e. What would your treatment policy be?
 A. Expectant, instruct the patient to call in case of deterioration

B. Refer to emergency department (ED) or outpatient clinic

C. Other + explanatory note

You will now receive the following vital signs from the wearable sensor:

*Heart rate 80 bpm, respiratory rate 12 bpm*

2f. What would your treatment policy be?
 A. Expectant, instruct the patient to call in case of deterioration

B. Refer to emergency department (ED) or outpatient clinic

C. Other + explanatory note

Instead of the previous vital signs, you will now receive the following vital signs from the wearable sensor:

*Heart rate 110 bpm, respiratory rate 12 bpm*

2g. What would your treatment policy be?
 A. Expectant, instruct the patient to call in case of deterioration

B. Refer to emergency department (ED) or outpatient clinic

C. Other + explanatory note

Instead of the previous vital signs, you will now receive the following vital signs from the wearable sensor:

*Heart rate 110 bpm, respiratory rate 20 bpm*

2h. What would your treatment policy be?
 A. Expectant, instruct the patient to call in case of deterioration

B. Refer to emergency department (ED) or outpatient clinic

C. Other + explanatory note

**Follow up of case 3**Patient was referred to the ED. A decline of 3 points in hemoglobin was seen, and an abdominal CT showed a hemoperitoneum. Patient underwent surgery, where lots of hematoma was removed and two bleedings were clipped. [End of case]

**Supplementary material 2: Patient criteria for same-day discharge metabolic bariatric surgery**

Eligibility for same-day discharge MBS was based on preoperative and postoperative criteria (before discharge).

The preoperative inclusion criteria:
- Age ≥18 years
- A residency within a 60-minute radius of [blinded] hospital [blinded]
- Have a have an adult caregiver present during the first postoperative night.

Preoperative exclusion criteria:
- Patients from psychiatric wards, inmates of prisons and other state institutions
- Insulin dependent diabetes
- Patients with active implants such as ICD and pacemaker
- Patients using a beta-blocker
- Patients diagnosed with OSA but without treatment

Postoperative criteria for same-day discharge:
- Body temperature <38.5°C
- Heart rate between 50 bpm and 100 bpm
- Respiratory rate between 5 and 25 bpm
- Oxygen saturation >94% and no previous desaturations
- No vomiting
- The ability to mobilize independently
- Numeric pain scale <4 (on scale of 10)
- A minimum intake of 150 mL orally and 1500 mL intravenously
- A postoperative decrease in hemoglobin of <1 mmol/L
- No administration of opioids in the 6 hours prior to discharge

Supplementary table 2: Median values of mean heart rate, respiratory rate, and activity level of patients with <50% missing data, with and without re-admission.

|  | **Readmitted (n=6)** | **Non-readmitted (n=67)** | **p-value** | **Effect size** |
| --- | --- | --- | --- | --- |
| Mean HR | 74.6 [71.7-81.8] | 70.6 [65.8-76.0] | 0.18 | -0.34 |
| Mean RR | 18.5 [18.2-20.2] | 17.6 [15.2-18.8] | 0.06 | -0.46 |
| Mean Activity | 3.3 [3.0-3.8] | 3.1 [2.9-3.4] | 0.28 | -0.27 |

HR: heart rate, RR: respiratory rate

Supplementary figure 1: Heart rate and respiratory rate data prior to readmission in six readmitted patients with >50% data coverage.

*
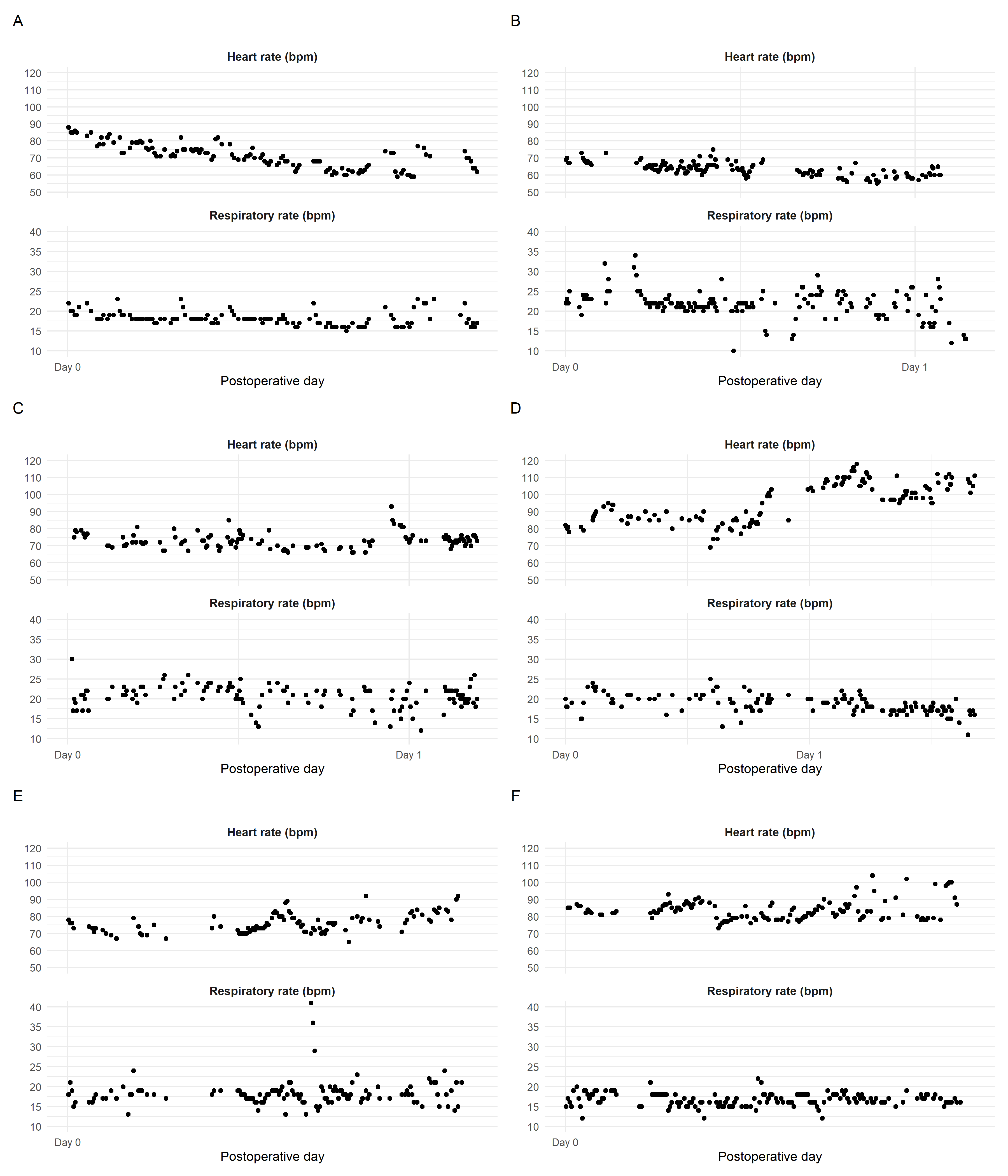
*
